# Editorial: Unraveling the associations between diet and mental health

**DOI:** 10.3389/fnut.2026.1823426

**Published:** 2026-03-26

**Authors:** Desirée Victoria-Montesinos, Teresa Balboa-Castillo, Rubén Fernández-Rodríguez, Miriam Garrido-Miguel, Estela Jiménez-López, Carla Soraya Costa Maia, Bruno Bizzozero-Peroni

**Affiliations:** 1 Faculty of Pharmacy and Nutrition, UCAM Universidad Católica San Antonio de Murcia, Murcia, Spain; 2 Department of Public Health, Universidad de La Frontera, Temuco, Chile; 3 EPICYN Research Centre of Cardiovascular and Nutritional Epidemiology, Universidad de La Frontera, Temuco, Chile; 4 Department of Physical Education and Sports, Faculty of Sports Science, Sport and Health University Research Institute (iMUDS), University of Granada, Granada, Spain; 5 Food and Mood Centre, Institute for Mental and Physical Health and Clinical Translation, Deakin University, Geelong, VIC, Australia; 6 The Grounded Minds Consortium, Deakin University, Melbourne, VIC, Australia; 7 Health and Social Research Center, Universidad de Castilla-La Mancha (UCLM), Cuenca, Spain; 8 Network for Research on Chronicity, Primary Care, and Health Promotion (RICAPPS), Cuenca, Spain; 9 Health Science Center, Universidade Estadual do Ceará, Fortaleza, CE, Brazil; 10 Instituto Superior de Educación Física, Universidad de la República, Maldonado, Uruguay; 11 Aging Research Center, Department of Neurobiology, Care Sciences and Society, Karolinska Institutet and Stockholm University, Stockholm, Sweden

**Keywords:** diet quality, dietary patterns, Mediterranean diet, mental health, nutritional psychiatry

Mental disorders (1) remain among the leading causes of disability worldwide, with depressive and anxiety disorders accounting for a substantial proportion of years lived with disability across all age groups. Over the past decade, the field of Nutritional Psychiatry has expanded rapidly, positioning diet and nutrition as fundamental to the prevention and management of mental disorders (2, 3). Across the lifespan and diverse global populations, a large and growing body of evidence supports the critical role of diet in the prevalence, management and risk of neurological and psychiatric disorders (4, 5). Diet is a ubiquitous exposure for the entire population, and diet quality is well-established as a modifiable factor for mental health at all ages (6). Despite notable advances in prevention and treatment, their prevalence has continued to rise over recent decades (7). This persistent increase highlights the need to identify modifiable determinants that can be addressed through both population-based and clinical strategies. Within this context, diet has emerged as a key lifestyle-related factor, influencing mental health through behavioral, metabolic, inflammatory, and neurobiological pathways (8).

The Research Topic “*Unraveling the associations between diet and mental health*” was conceived to integrate current evidence on how dietary exposures, patterns, and eating behaviors relate to mental health outcomes across different life. It brings together 14 contributions, including original research articles, cross-sectional epidemiological studies, population-based analyses, and narrative reviews. Together, these studies span diverse geographical settings, age groups, and methodological approaches within the field of nutritional epidemiology, offering a broad and integrated view of the diet-mental health relationship.

A central theme across several contributions is the association between dietary patterns, diet quality, and depressive or anxiety-related outcomes. Using an inflammatory-diet framework, You and Xia examine the relationship between dietary inflammatory potential and mental health outcomes, reinforcing inflammation as a plausible biological pathway linking diet to psychological distress. In a complementary line of research, Zhao et al. analyze the impact of private caregivers on nutritional risk and anxiety in stroke survivors with dysphagia, illustrating how care-related factors intersect with nutritional status and psychological wellbeing in a vulnerable clinical population.

Within this same framework, Ding and Zou assess the Prognostic Nutritional Index as an integrative marker of nutritional and inflammatory status in individuals with depressive symptoms. Their findings highlight the potential utility of composite nutritional indicators for risk stratification and prognosis in mental health research. Extending dietary pattern research toward sustainability-oriented models, Tabatabaei et al. investigate mental health outcomes in relation to adherence to the EAT-Lancet dietary pattern, linking planetary health-aligned diets with psychological wellbeing and reinforcing the public health relevance of sustainable nutrition frameworks.

Beyond dietary patterns themselves, several contributions emphasize the importance of eating behaviors and emotional processes in shaping mental health outcomes. In a census-matched study of Czech young adults, Poslt Königová et al. suggest that depressive and anxiety symptoms, together with cognitive emotion-regulation strategies, are differentially associated with overall diet quality and specific eating behavior phenotypes. These findings highlight that dietary choices cannot be fully understood without considering emotional and cognitive mechanisms, and they support the integration of psychological components into nutritional prevention and intervention strategies.

At the population level, the influence of contemporary food environments is addressed by Bala et al., who provide large-scale evidence linking frequent ultra-processed food consumption with poorer mental functioning and increased psychological distress across multiple countries. By situating individual dietary behaviors within broader structural and environmental contexts, this study strengthens arguments for policy-level approaches to mental health promotion and prevention.

Beyond the food environment itself, the interaction between diet and other lifestyle-related behaviors emerges as another recurring theme within this Research Topic. Jiazhi et al. show that physical activity is associated with lower levels of depression, anxiety, and stress among college students, both directly and indirectly through healthier dietary behaviors. In clinical and community samples, Wu et al. and Gillespie et al. further examine how specific nutritional exposures and diet quality indices relate to depressive and anxiety symptoms, reinforcing the notion that diet should not be examined in isolation, but rather as part of a broader lifestyle pattern when addressing mental health outcomes.

Mechanistic insights are further provided through contributions grounded in nutritional psychiatry and the gut-brain axis. In a narrative review, Horovitz synthesizes evidence linking inflammatory bowel disease, diet, gut microbiota, and psychiatric comorbidities, illustrating how chronic inflammation and dysbiosis may contribute to anxiety and depressive symptoms. Complementing this perspective, Hunter et al. present original data on nutrient insufficiencies and symptom severity in children and adults with ADHD and other neurodivergent conditions, emphasizing the relevance of brain-selective nutrients and individualized nutritional approaches.

Additional contributions broaden the scope of the Research Topic by examining mental health outcomes in relation to dietary exposures across diverse populations and contexts. Nielsen et al. offer a psychological perspective by situating dietary behaviors and food-related experiences within broader psychosocial and cognitive frameworks, including the role of food sensitivities and distressing dream experiences. Yan et al. explore how dietary magnesium intake modifies the association between vitamin A intake and depression risk in a large, nationally representative adult sample, highlighting sex-specific nutrient-nutrient interactions of relevance for precision nutrition. Koc et al. investigate links between hedonic hunger, diet-related behaviors, obesity, and health-related quality of life in adolescents, underscoring the importance of reward-driven eating processes in youth mental health and wellbeing.

Taken together, the 14 contributions included in this Research Topic converge on several key messages. Diet quality, dietary patterns, and levels of food processing show consistent associations with mental health outcomes, while emotional regulation, lifestyle behaviors, inflammation, and gut-brain mechanisms appear to mediate or modify these relationships. At the same time, the predominance of cross-sectional designs underscores the need for longitudinal and interventional research to strengthen causal inference.

In conclusion, this Research Topic highlights diet as a central and modifiable component of mental health promotion and prevention across the life course. Integrating nutritional strategies with psychological, behavioral, and public health approaches may provide a valuable opportunity for reducing the global burden of mental disorders and improving mental wellbeing at both individual and population levels.
